# Predictive Factors for Cut-Out Risks of Unstable Trochanteric Fractures

**DOI:** 10.7759/cureus.74355

**Published:** 2024-11-24

**Authors:** Sergiu Iordache, Adrian Cursaru, Mihai Aurel Costache, Razvan Spiridonica, Gafita Elena, Lana Jiroudi, Raluca Cursaru

**Affiliations:** 1 Orthopaedics and Traumatology, University Emergency Hospital, Bucharest, ROU; 2 General Medicine, Carol Davila University of Medicine and Pharmacy, Bucharest, ROU; 3 Diabetes and Endocrinology, National Institute of Diabetes, Nutrition and Metabolic Diseases-Prof. N. Paulescu, Bucharest, ROU

**Keywords:** cervicocephalic screw, cut-out, diabete mellitus, risk factors, trochanteric fractures

## Abstract

Fractures of the trochanteric mass represent a significant proportion of hip fractures. These fractures often occur in the elderly due to compromised bone quality, leading to a high predisposition for instability at the fracture site.

The study was conducted through a retrospective analysis of 1,259 hospitalizations in the Department of Orthopedics and Traumatology of the Bucharest University Emergency Hospital between 2022 and 2023, including patients with various types of trochanteric mass fractures: basicervical, per trochanteric, intertrochanteric, subtrochanteric, and trochanter-diaphyseal fractures. In the selection process, 59 patients who met the specific inclusion criteria for the study were chosen. The study population included 59 patients selected based on the afore-mentioned inclusion and exclusion criteria. For these patients, the demographic and clinical data revealed the following: the age of the patients varied between 26 and 91 years, with a mean of 74.69 years, a median of 80 years, and a standard deviation of 14.94 years. The first quartile was at 71 years, and the third quartile was at 84.5 years, resulting in an interquartile range of 13.5 years.

The article aims to study prognostic factors for the risk of cut-out in the surgical treatment of unstable trochanteric fractures. A systematic evaluation of patient characteristics, fracture types, and surgical treatment options could establish the relevant factors in predicting the occurrence of cut-out. The results of this research have the potential to facilitate clinical decision-making, optimize treatment strategies, and improve outcomes for patients with unstable trochanteric fractures undergoing surgery.

## Introduction

Fractures of the trochanteric mass represent a significant proportion of hip fractures. These fractures often occur in the elderly due to compromised bone quality, leading to a high predisposition for instability at the fracture site. The importance of unstable trochanteric fractures lies in their association with functional impairment, prolonged hospitalization, and high mortality rates, making them an urgent public health concern [1-2].

Surgical intervention is commonly used to stabilize unstable trochanteric fractures and facilitate early mobilization and rehabilitation. One of the most feared complications of surgical treatment is the occurrence of cut-out, characterized by the migration of fixation devices from their intended position into the femoral head. Cut-out compromises fracture stability, leading to implant failure, loss of reduction, and poor functional outcomes. Additionally, the migration of implants into the soft tissues or adjacent joint spaces can cause considerable morbidity and may necessitate further surgical interventions [3-4].

Given the potential consequences of cut-out in the surgical treatment of unstable trochanteric fractures, there is an urgent need to identify prognostic factors associated with this complication. Understanding the factors that predispose patients to a higher risk of the cut-out is essential for achieving a desired goal, such as adapting treatment strategies and selecting the most suitable surgical techniques and implants based on the type of fracture and the patient’s risk profiles [5-6].

## Materials and methods

This study was conducted through a retrospective analysis of 1,259 admissions to the Orthopedics and Traumatology Department of the Bucharest University Emergency Hospital between 2022 and 2023. It included patients diagnosed with various types of trochanteric mass fractures: basicervical, per trochanteric, intertrochanteric, subtrochanteric, and trochanter-diaphyseal fractures. In the selection process, 59 patients who met the specific inclusion criteria for the study were selected.

Inclusion and exclusion criteria

The inclusion criteria involved adult patients aged over 18, in accordance with the hospital’s profile for adult patient treatment, diagnosed with unstable trochanteric fractures and surgically treated with intramedullary nails featuring a cervicocephalic screw (gamma nail). Selection also depended on the availability of complete and accessible data regarding the variables of interest, including age, sex, presence of hypertension, osteoporosis, diabetes mellitus, fracture stability, nail positioning, fracture reduction, and the presence of cut-out.

As for the exclusion criteria, patients with stable trochanteric fractures or fractures on pathological bone, those with severe comorbidities, those who did not follow the standardized treatment protocol, those with a history of surgeries in the same anatomical region, and those for whom data from the sources used for analysis were incomplete or ambiguously defined were excluded. These criteria were implemented to ensure the consistency and relevance of the collected data, eliminating variables that could potentially distort the analyses performed.

Statistical analysis

Statistical analysis was performed to identify significant prognostic factors for the risk of cut-out in the treatment of unstable trochanteric fractures using intramedullary implants such as gamma nails. The analysis included multiple steps and various statistical methods adapted to the specifics of the collected data and the study objectives.

Python (Python Software Foundation, https://www.python.org/) and R Studio (Posit, http://www.rstudio.com/) programming languages were used to process demographic, clinical, and surgical data (including variables such as gamma nail positioning, fracture reduction, and the presence of cut-out). In Python, statistical libraries such as pandas, numpy, scipy, and statsmodels were employed, while in R Studio, statistical packages like ggplot2, dplyr, and caret were utilized. The use of these softwares allowed efficient data analysis and the application of advanced statistical analyses.

Statistical analyses performed included descriptive analyses to summarise patients’ demographic and clinical data using descriptive statistics (means, medians, standard deviations, frequencies). Turkey’s test was also applied to assess age differences between males and females, identifying statistically significant differences between groups. Logistic regression models were used to evaluate the cumulative influence of multiple factors on cut-out risk. This method allowed for the identification of the factors that contribute the most to the occurrence of cut-out. Following the collection of data from 59 patients who underwent intramedullary fixation surgery for unstable trochanteric fractures, the following 10 variables were used (Table 1).

**Table 1:** Variables collected in the study.

Variable	Data type	Description
Age	Numeric	Patients' age in years
Diabetes mellitus	Categorical	Presence or absence of diabetes mellitus
Hypertension	Categorical	Presence or absence of hypertension
Osteoporosis	Categorical	Presence or absence of osteoporosis
Stability	Categorical	Fracture stability (stable or unstable)
Reduction	Categorical	Quality of fracture reduction (anatomical or functional)
Positioning	Categorical	Nail positioning (lower third, middle third, or upper third)
Cut-out	Categorical	Presence or absence of cut-out
Name	Character	Patients' names
Sex	Categorical	Patients' gender (male or female)

## Results

The study population included 59 patients selected based on the aforementioned inclusion and exclusion criteria. The demographic and clinical data for these patients revealed the following: their ages ranged from 26 to 91 years, with a mean age of 74.69 years, a median of 80 years, and a standard deviation of 14.94 years. The first quartile was at 71 years, and the third quartile was at 84.5 years, resulting in an interquartile range of 13.5 years. The sex distribution indicated that 21 patients (35.59%) were male, while 38 patients (64.40%) were female.

Age differences between males and females were analyzed using Tukey’s test within a linear regression ANOVA model. The results showed that the mean age of females was 79.97 years, whereas the mean age of males was 65.14 years. The average age difference between males and females was 14.83 years, with a 95% confidence interval ranging from -22.03 to -7.63 years. The adjusted p-value was 0.0001223, indicating a statistically significant difference.

In terms of comorbidities, 39 patients (66.10%) had hypertension, while 20 patients (33.89%) did not have this condition. Additionally, 35 patients (59.32%) were diagnosed with osteoporosis, whereas 24 patients (40.67%) did not present this condition. Regarding diabetes mellitus, 15 patients (25.42%) were diagnosed with the disease, while 44 patients (74.57%) did not have diabetes.

According to the collected data, 43 patients (72.88%) underwent anatomic reduction, while 16 patients (27.11%) received functional reduction. Concerning the positioning of the gamma nail, it was placed in the lower third for 27 patients (45.76%), in the middle third for 30 patients (50.84%), and in the upper third for 2 patients (3.38%). All fractures included in the study were unstable (59 patients, 100%). As for the incidence of cut-out, it was present in 4 patients (6.77%) and absent in 55 patients (93.22%).

To investigate the risk of cut-out among patients, biomarker analysis was utilized. Although this method is commonly used for screening or diagnosis, it can also be applied for prognostic purposes, providing additional insights that traditional methods may not offer. Furthermore, Receiver Operating Characteristic (ROC) curves were employed, a well-established method for evaluating the accuracy of tests involving continuous or ordinal variables.

In this study, the influence of the following variables on the risk of cut-out was analyzed: patient age, nail positioning, presence of osteoporosis, the combined influence of nail positioning and osteoporosis, fracture reduction, hypertension, and diabetes. Additionally, the combined influence of these factors on the risk of cut-out will be examined. The ROC function enables the development of a mathematical model for each variable and combination of variables, with an optimal AUC (area under the curve) score representing the overall ability of the test to distinguish between positive and negative cases, expected to be between 80% and 90%.

Age

When modeling the risk of cut-out among patients as a function of age, an AUC of 45.45% was obtained, with a 95% confidence interval ranging from 11.3% to 79.61% (DeLong). Although this result exceeds the 45% threshold necessary to suggest a potential causal relationship, its minimal level indicates the need to explore additional, more relevant factors.

The calculation of the optimal point between specificity (the proportion of correctly identified positive cases) and sensitivity (the proportion of correctly identified negative cases), known as the “threshold,” for age revealed that the optimal value is 72.5 years, with a sensitivity of 50% and a specificity of 67.27%. Using a generalized linear model (GLM) with the cut-out variable having a binomial distribution, a p-value of 0.553 was obtained (p>0.05), indicating that the model is not sufficiently significant to explain the data. Additionally, the Akaike Information Criterion (AIC) had a relatively low value of 32.82, suggesting a modest fit for the model.

**Figure 1:**
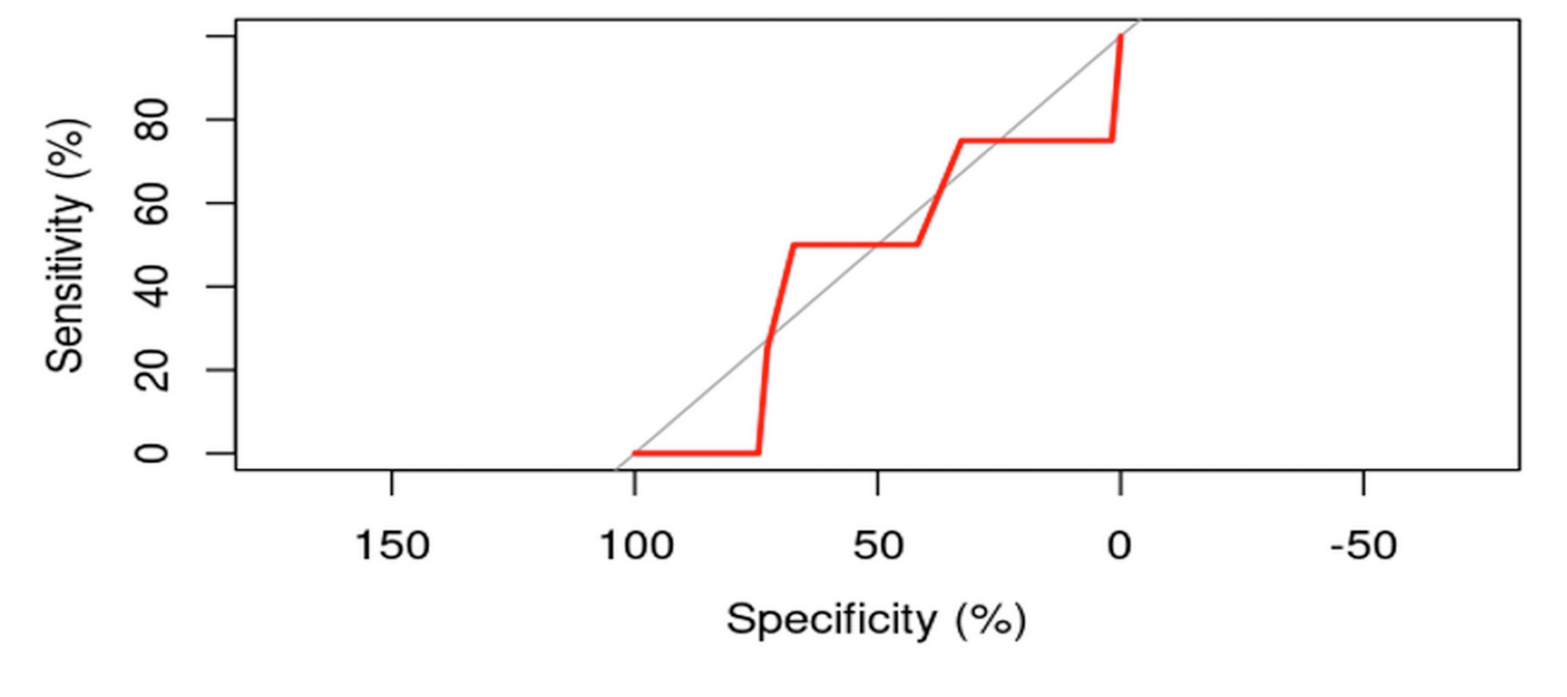
ROC curve for evaluating the influence of age on the risk of cut-out.

It is observed that the ROC curve is not predominantly above the 45-degree diagonal, further suggesting the necessity of identifying another, more appropriate causal factor (Figure 1).

Although the obtained p-value is greater than 0.05, the coefficients from the logistic regression model can be used to calculate the probability of cut-out occurrence. In the context of logistic regression, the coefficients are utilized to compute the ratio between the probability of an event occurring and the probability of it not occurring, known as the odds ratio (OR). Calculating individual values, the average probability of cut-out occurrence is p=0.06780. 

The analysis of individual values for the probability of cut-out occurrence reveals significant variability. The minimum and maximum probable values suggest that while some patients exhibit a low risk (min. 0.01639), others may have a relatively higher risk (max. 0.09714). The average probability of 0.06780 indicates a generally low risk in the studied population, but the wide distribution of values underscores the importance of identifying other risk factors that could significantly influence the occurrence of cut-out.

Gender

In the logistic regression model, gender was not identified as a statistically significant factor influencing cut-out risk (p = 0.994). While the gender coefficient was highly negative (-17.4260), indicating a potential effect, this outcome is not strongly supported due to the small sample size, with only 4 female patients presenting cut-out. Although the model demonstrated a good overall fit with a low AIC value (29.574), the extreme coefficient, alongside the lack of statistical significance, suggests that no clear relationship between gender and cut-out risk can be confirmed based on this dataset.

Positioning of the nail

The ROC curve analysis for nail positioning yielded an AUC of 45.91%, with a 95% confidence interval ranging from 8.421% to 83.4% (DeLong). This result suggests that nail position may be considered a factor in the occurrence of cut-out; however, its precise influence will need further evaluation.

**Figure 2:**
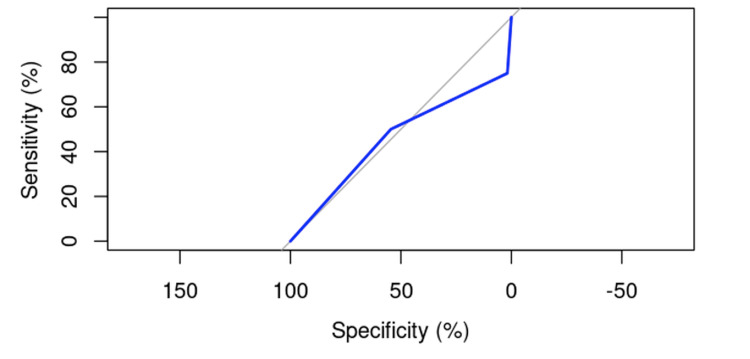
ROC curve for evaluating the influence of nail positioning on the risk of cut-out.

A logistic regression model was performed to analyze the relationship between nail position and cut-out, resulting in a p-value of 0.5231, indicating a lack of statistical significance (p > 0.05). Nonetheless, the ROC curve analysis visually suggests that there may be an influence of nail positioning on the incidence of cut-out (Figure 2). The variance in the probability of cut-out occurrence is significant, indicating a substantial contribution to the risk influence.

Osteoporosis

**Figure 3:**
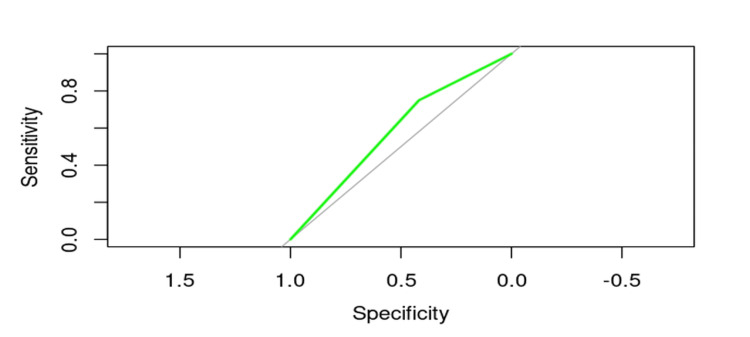
ROC curve evaluating the influence of osteoporosis on the risk of cut-out.

The ROC curve analysis demonstrated that the influence of osteoporosis on the risk of cut-out is significant, with an AUC value of 58.41%. This result suggests that osteoporosis is an important factor in the occurrence of cut-out. Although the logistic regression model indicated a nonsignificant p-value of 0.51723, the ROC curve clearly illustrates the impact of osteoporosis on this risk (Figure 3).

To calculate the probability of risk p using the model coefficients, the following descriptive values were obtained (Table 4). These values suggest that the variability in the probability of cut-out occurrence is significantly influenced by the presence of osteoporosis. The mean probabilities and extreme values indicate that osteoporosis has a considerable impact on the risk of cut-out, underscoring the need for more in-depth analysis to fully understand this factor.

Combined Influence of nail positioning and osteoporosis on cut-out risk

To assess the combined influence of nail positioning and osteoporosis on the risk of cut-out, a logistic regression model was created that includes both factors. The results indicated p-values of 0.4762 for nail positioning and 0.4716 for osteoporosis. Although these values do not reach the threshold for statistical significance, they suggest that each factor contributes similarly to the risk of cut-out occurrence.

**Figure 4:**
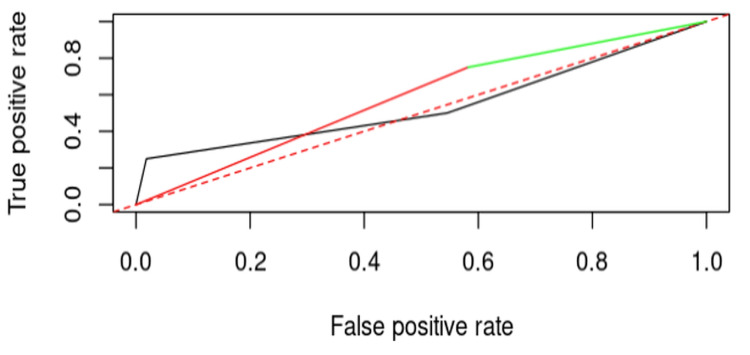
Combined influence of nail positioning and osteoporosis on the risk of cut-out.

The ROC curve illustrates the distribution of influence for each factor. Utilizing the performance function in this analysis allowed for the determination of the optimal point between sensitivity and specificity, highlighting how the combination of these two factors affects the probability of cut-out (Figure 4).

Fracture reduction

**Figure 5:**
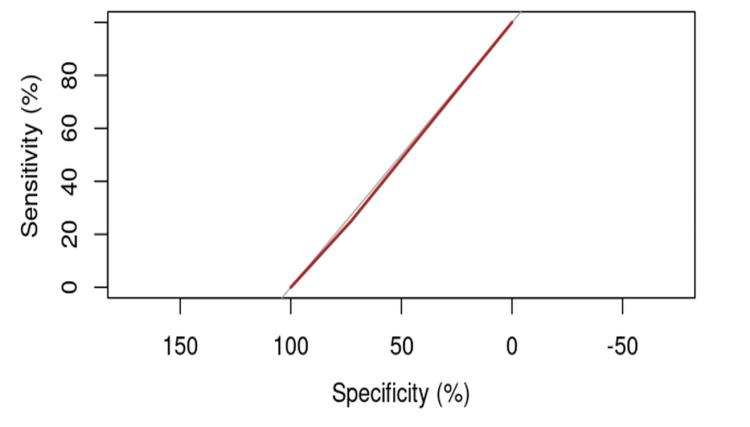
ROC curve for evaluating the influence of fracture reduction on the risk of cut-out.

The analysis of cut-out risk based on fracture reduction revealed a p-value of 0.921, significantly higher than that of other studied factors, indicating low statistical significance. The AIC value was 33.243, while the AUC reached 48.86%, suggesting a modest predictive capacity (Figure 5).

The probability of cut-out occurrence, determined based on fracture reduction, is presented in the tables below. These data reflect a low variability in the probability of cut-out, suggesting that fracture reduction has a limited impact on the risk of its occurrence.

Hypertension

**Figure 6:**
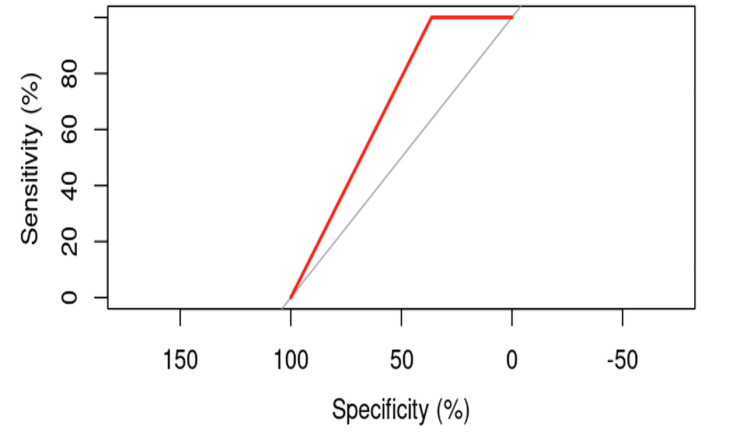
ROC curve for assessing the influence of hypertension on the risk of cut-out.

Hypertension demonstrated the highest AUC among all analyzed factors, reaching a value of 68.18% (Figure 6). This result suggests that hypertension is an important predictor of the risk of cut-out.

The probability of cut-out occurrence based on the presence of hypertension was calculated using the model. The wide variability of these values suggests a considerable influence of hypertension on the risk of cut-out.

Diabetes mellitus

**Figure 7:**
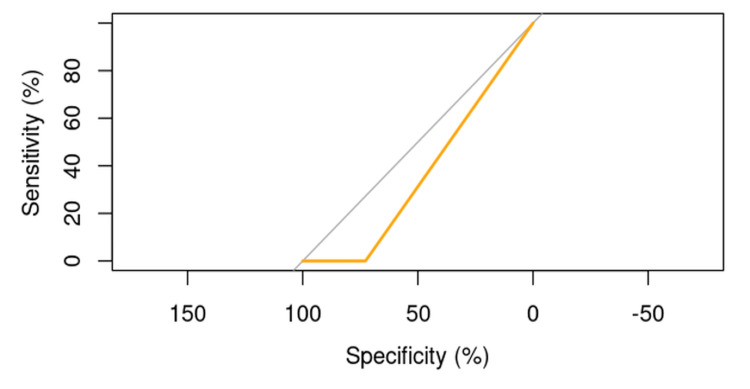
ROC curve for evaluating the influence of diabetes on the risk of cut-out.

Diabetes mellitus presents a p-value of 0.995 and an AIC value of 30.808, indicating an insignificant influence in predicting the risk of cut-out according to the AUC, which is 36.36%. However, further analysis of the logistic regression model reveals that diabetes mellitus has the highest weight in the risk of cut-out, with a coefficient of -18.66589 (Figure 7).

The variability of the probability of cut-out occurrence for diabetes is nearly similar to that of hypertension, being surpassed only by the variability associated with the positioning of the nail. Stability analysis was not possible due to the exclusive presence of values of 0.

Combined influence of factors on the risk of cut-out

To evaluate the combined influence of various factors on the risk of cut-out, a generalized linear model (GLM) was performed. The variables included in the model were nail position, fracture reduction, osteoporosis, diabetes, and age (Table 2).

**Table 2:** Logistic regression model for individual factors.

Variables	p-value
Intecept	0.152
Screw	0.331
Reduction	0.74
Osteoporosis	0.797
Diabetes Mellitus	0.995
Age	0.514

The detailed results are recorded in Table 3, which includes hypertension in the model. The best p-value was obtained for nail positioning (0.397), followed by fracture reduction (0.703), although neither of these values is statistically significant.

**Table 3:** Logistic regression model including hypertension. Pr(>IzI): p-value of z parameter.

Coefficient	Estimate	Standard error	z value	Pr(>IzI)
Intercept	-23.71	3821.938	-0.006	0.995
Screw	0.95	1.12	0.84	0.39
Reduction	0.45	1.43	0.38	0.70
Osteoporosis	0.35	1.73	0.20	0.83
Diabetes mellitus	-18.66	4402.73	-0.004	0.99
Age	0.01	0.065	0.18	0.85
Hypertension	18.55	3821.93	0.005	0.99

However, diabetes has a coefficient of 18.665, and hypertension has a coefficient of 18.553, indicating their strong influence on the risk of cut-out. To formulate robust conclusions, the cumulative probability of cut-out occurrence was calculated, integrating all factors, with the mean value p=0.0678, along with the following descriptive statistics (Table 4).

**Table 4:** Probability of cut-out.

Age differences between males and females	Comparison	Mean difference	95% Confidence interval	Adjusted p-value	Mean difference	
	M-F	-14.830	-22.031 to -7.6298	0.000122		
Probability of cut-out occurrence by age	Min	1st Quartile	Median	Mean	3rd Quartile	Max
	0.016	0.058	0.073	0.067	0.082	0.097
Probability of cut-out occurrence based on nail positioning	Min	1st Quartile	Median	Mean	3rd Quartile	Max
	0	0.012	0.063	0.067	0.116	0.208
Probability of cut-out occurrence based on osteoporosis	Min	1st Quartile	Median	Mean	3rd Quartile	Max
	0.0416	0.0416	0.0857	0.067	0.116	0.085
Probability of cut-out occurrence based on fracture reduction quality	Min	1st Quartile	Median	Mean	3rd Quartile	Max
	0.062	0.062	0.069	0.067	0.069	0.069
Probability of cut-out occurrence based on hypertension	Min	1st Quartile	Median	Mean	3rd Quartile	Max
	0	0	0.102	0.067	0.102	0.102
Probability of cut-out occurrence based on diabetes	Min	1st Quartile	Median	Mean	3rd Qu.	Max
	0	0.045	0.045	0.067	0.090	0.090
Cumulative probability of cut-out occurrence for all factors	Min	1st Quartile	Median	Mean	3rd Quartile	Max
	0	0	0	0.067	0.146	0.293

**Figure 8:**
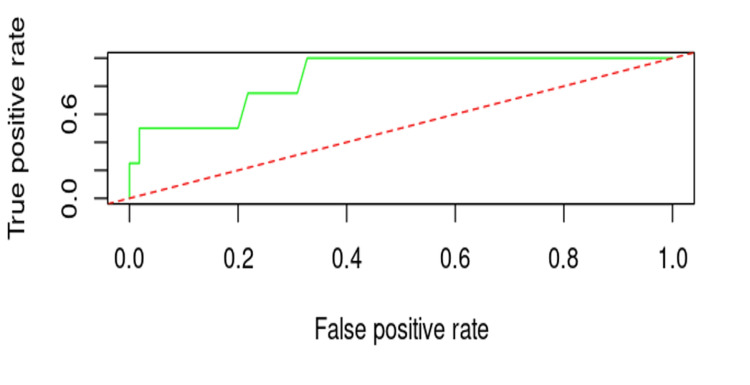
ROC curve for prognosis of cut-out occurrence based on the combined influence of factors.

The ROC curve illustrates the prognosis of cut-out occurrence based on the combined influence of nail positioning, fracture reduction, osteoporosis, diabetes, age, and hypertension, with an AUC of 0.864. This result demonstrates that the combined regression model has superior predictive capacity compared to each individual factor (Figure 8).

## Discussion

The results of this study indicate that age is not a significant predictor of cut-out risk in the treatment of unstable trochanteric fractures, which is consistent with other studies that have reached similar conclusions about the lack of direct influence of age on cut-out risk [7-10].

The analysis also showed no statistically significant difference in cut-out risk between males and females, although it should be noted that all cases of cut-out in the sample were female. Due to the small sample size, definitive conclusions about gender as a risk factor are limited. This finding aligns with previous research, such as that by Hsueh et al. (2010), which reported no significant gender differences when controlling for clinical and biomechanical factors [11].

In contrast, hypertension was shown to significantly impact cut-out risk. According to a study by Do Carmo et al. (2020), hypertension contributes to bone fragility and can increase the risk of postoperative complications by affecting bone microcirculation and slowing the healing process. This is further supported by research demonstrating that hypertension negatively influences bone mineral density, subsequently increasing the risk of fractures and postoperative complications [12].

The analysis in this study, indicating a negative regression coefficient for diabetes mellitus (18.665), suggests a protective effect against the risk of cut-out in unstable trochanteric fractures. These findings are unusual in the context of general literature, which typically associates diabetes with an increased risk of postoperative complications. However, other studies have reported similar results. For example, patients with type 2 diabetes may have bone mineral density (BMD) similar to or higher than those without diabetes, although their fracture risk may be elevated due to other factors [13]. Additionally, another study found that diabetes is not a significant predictor of postoperative complications in certain surgical contexts. The biological explanation for this negative regression coefficient may be related to metabolic changes in bone in diabetic patients. Although diabetes is associated with reduced bone quality and increased fracture risk, treatments for diabetes, such as SGLT2 inhibitors and metformin, may have beneficial effects on bone health [14-15].

The impact of various factors on the risk of cut-out in orthopedic procedures, particularly focusing on osteoporosis and nail positioning, It indicates that, although the p-value related to osteoporosis's influence on cut-out risk did not achieve statistical significance, the area under the curve (AUC) and variance in probabilities suggest that osteoporosis may contribute to weakened implant stability and an increased risk of cut-out. These conclusions are supported by a study conducted by Fink et al. (2024), which demonstrated that patients with severe osteoporosis have a significantly higher risk of postoperative complications, including cut-out [16]. Additionally, the importance of proper positioning of the cervicocephalic nail is highlighted. Despite a p-value of 0.5231 indicating no clear statistical significance, ROC curve analysis and the notable variance in probabilities suggest that nail positioning is crucial for minimizing cut-out risk. The study by Goffin et al. (2013), who noted that suboptimal nail positioning can lead to implant instability and migration, thereby increasing the risk of cut-out [7].

In contrast, the results indicate that fracture reduction does not significantly influence cut-out risk. A study by Bojan et al. (2022) supports this conclusion, stating that while anatomical reduction is beneficial for long-term stability, it is not a decisive factor in preventing cut-out. Overall, the findings emphasize the complex interplay among osteoporosis, nail positioning, and fracture reduction in relation to cut-out risk, suggesting areas for further research and clinical focus.

The generalized logistic regression model (GLM) showed that the combined influence of variables provides superior predictive capacity for cut-out risk, confirmed by an AUC of 0.864, which is significantly higher than the results obtained through the individual analysis of the variables discussed earlier. These results are comparable to those obtained in the study by Bojan et al. (2022), which emphasizes the importance of a multifactorial analysis in predicting cut-out [17].

Limitations of the study

This study is not without limitations that warrant consideration. The modest sample size may constrain the generalizability of the findings, as a larger cohort would enhance the strength and scope of the conclusions. Moreover, the retrospective design, while informative, introduces inherent biases related to data completeness and selection. Factors such as the nutritional status of patients, their medication history, and smoking habits were not examined, though these may hold relevance for bone health and recovery outcomes. Moreover, the single-center nature of the study may limit its broader applicability, as variations in clinical practices and patient populations could influence the results. The reliance on radiological assessments of osteoporosis, without the precision of DEXA scanning, and the absence of the Tip-Apex Distance (TAD) as a metric for nail positioning may also have impacted the accuracy of certain conclusions.

Future directions

To advance the understanding of cut-out risk in unstable trochanteric fractures, future research should expand into larger, multicenter studies, which would provide more reliable and broadly applicable insights. Prospective methodologies would enhance data accuracy and reduce inherent biases, ensuring stronger, more reliable conclusions. Exploring under-examined factors, such as patient nutrition, specific medications, and lifestyle behaviors, could unveil additional predictors of surgical outcomes. The integration of more precise diagnostic tools, like DEXA for assessing bone density, along with the application of standardized parameters such as the Tip-Apex Distance (TAD) for implant positioning, would sharpen risk assessments. Moreover, incorporating psychosocial dimensions, including familial and emotional support, could yield a more holistic view of the factors that influence recovery and long-term patient outcomes.

## Conclusions

This study highlights the potential impact of clinical factors such as hypertension and osteoporosis on the risk of cut-out in the surgical treatment of unstable trochanteric fractures using gamma nails. While the positioning of the cervicocephalic screw seems to play an important role in reducing this risk, factors such as age and gender may have subtler effects that warrant further exploration. A multifactorial approach remains essential for optimizing treatment strategies and improving clinical outcomes. However, additional research is needed to better understand the complex interactions between these variables and to refine the prediction of cut-out risk.
